# The association of birth order with later body mass index and blood pressure: a comparison between prospective cohort studies from the United Kingdom and Brazil

**DOI:** 10.1038/ijo.2013.189

**Published:** 2013-10-29

**Authors:** L D Howe, P C Hallal, A Matijasevich, J C Wells, I S Santos, A J D Barros, D A Lawlor, C G Victora, G D Smith

**Affiliations:** 1 MRC Integrative Epidemiology Unit at the University of Bristol, Bristol, UK; 2 School of Social and Community Medicine, University of Bristol, Bristol, UK; 3 Postgraduate Programme in Epidemiology, Federal University of Pelotas, Pelotas, Brazil; 4 Institute of Child Health, University College London, London, UK

**Keywords:** ALSPAC, birth order, blood pressure, body mass index, cardiovascular, Pelotas, siblings

## Abstract

**Background:**

Previous studies have found greater adiposity and cardiovascular risk in first born children. The causality of this association is not clear. Examining the association in diverse populations may lead to improved insight.

**Methods:**

We examine the association between birth order and body mass index (BMI), systolic and diastolic blood pressure (SBP/DBP) in the 2004 Pelotas cohort from southern Brazil and the Avon Longitudinal Study of Parents and Children (ALSPAC) from Bristol, south-west England, restricting analysis to families with two children in order to remove confounding by family size.

**Results:**

No consistent differences in BMI, SBP or DBP were observed comparing first and second born children. Within the Pelotas 2004 cohort, first born females were thinner, with lower SBP and DBP; for example, mean difference in SBP comparing first with second born was −0.979 (95% confidence interval −2.901 to 0.943). In ALSPAC, first born females had higher BMI, SBP and DBP. In both cohorts, associations tended to be in the opposite direction in males, although no statistical evidence for gender interactions was found.

**Conclusions:**

The findings do not support an association between birth order and BMI or blood pressure. Differences to previous studies may be explained by differences in populations and/or confounding by family size in previous studies.

## Introduction

The association between smaller birth size and greater cardiovascular risk has been well documented.^1, 2, 3^ However, as this association is observed across the whole range of birth weights, rather than being a threshold whereby only those of low birth weight are at increased risk, it has been suggested that a wide range of factors other than birth size may be important.^4^

First born children tend to be of lower birth weight than subsequent children born to the same mother; for example, one Norwegian study found an average of 200 g increase in birth weight between first and second born children.^5^ There is evidence from some studies that being a first born child is associated with more adverse body composition and cardiovascular risk. Analysis of the 1982 and 1993 birth cohorts from Pelotas, Brazil has demonstrated that first born children are born smaller, but grow faster in infancy and are more adipose, with a more adverse cardiovascular profile in late adolescence/early adulthood.^4, 6^ Further studies in India^7^ and the Philippines^8^ have also demonstrated greater adiposity in first born children. Most of the evidence supporting the hypothesis that first born children are at greater cardiovascular risk has come from low- and middle-income countries; studies in higher income countries have had more mixed findings.^9, 10, 11, 12, 13, 14^

It is hypothesised that the combination of small size at birth and rapid growth in infancy could be the mechanism through which birth order influences cardiovascular risk.^4^ However, in addition to biological ecahnisms, social causation is also possible. Caring for, sharing with and competing with other siblings have been postulated as an explanation for proposed personality differences by birth order;^15^ these factors might explain the relationship between birth order and cardiovascular risk.

In this manuscript, we examine differences between first and second born children in terms of birth weight and length, later height, weight, body mass index (BMI), systolic blood pressure (SBP) and diastolic blood pressure (DBP) in two cohorts. First, we explore the 2004 cohort from Pelotas, a city in the South of Brazil. Associations of birth order with birth size, early growth and cardiovascular health have previously been published in the cohorts who were born in this city in 1982 and 1993.^4, 6^ There is a 22-year span between the first Pelotas cohort and the 2004 cohort we analyze here; during this time, the socio-economic environment and family patterns have changed markedly. We also examine these associations in the Avon Longitudinal Study of Parents and Children (ALSPAC), a cohort study from the United Kingdom, where social differences between first and second born children are likely to be less stark than those in Pelotas. Comparing the association between birth order and cardiovascular risk between cohorts in different environments may help us to understand the consistency of the association between diverse settings and provide clues as to the underlying mechanisms.

## Materials and Methods

The Pelotas 2004 cohort attempted to recruit all births from mothers’ resident in the urban area of the city of Pelotas, Southern Brazil, between 1/1/2004 and 31/12/2004 inclusive. Births were identified by daily visits to the five maternity hospitals. In the city of Pelotas, more than 99% of all deliveries take place in hospitals. In 2004, of the 4263 live births born to mothers living in the urban area of the city of Pelotas, 4231 were included in the prenatal study (0.8% loss) and were enrolled in the cohort study. Further information about the methodology of the 2004 Pelotas birth cohort study is described in detail elsewhere.^16, 17^

ALSPAC is a prospective cohort study in South-West England.^18, 19^ Pregnant women resident in one of three Bristol-based health districts with an expected date of delivery between 1 April 1991 and 31 December 1992 were invited to take part in the study. Of these women, 14 541 were recruited. From these pregnancies, there were 14 062 live-born children, 13 988 of whom were alive at 1 year. The study website contains details of available data through a fully searchable data dictionary (http://www.bris.ac.uk/alspac/researchers/data-access/data-dictionary/). Ethical approval for the study was obtained from the ALSPAC Law and Ethics Committee and the Local Research Ethics Committees.

### Defining birth order

In order to control for confounding by parity/family size, we restricted all analyses to families in which only two pregnancies resulted in births (live births and still births were all included, although sensitivity analyses demonstrated that results were unchanged if still births were excluded). Therefore, our sample consists of some participants who are the first of two children for their mother and some who are the second of two children. Miscarriages and terminations were not counted for the purposes of our study, as the vast majority of them will have occurred early in gestation and are unlikely to have resulted in major change to either the parenting received by the child (proposed social mechanism) or the mother’s vascular system (proposed biological mechanism).

Number of pregnancies and their outcomes before the index child was ascertained from questionnaires completed by the mother at recruitment into the cohort studies. Multiple questionnaires completed by the mother after the birth of the index child (up to the time of assessment of offspring outcomes relevant to this study in ALSPAC, but only up to age 4 in Pelotas since questions about siblings were not asked after this time) were used to identify subsequent pregnancies and their outcomes.

### Measurements

In the Pelotas 2004 cohort, birth weight and length were measured within 24 h of delivery. Bbirth weight was measured by hospital staff with 10-g precision pediatric scales that were regularly calibrated by the research team. Supine length measurements were taken by trained research fieldworkers using AHRTAG infant meters (AHRTAG baby length measures, London). At a research clinic visit when participants were ∼7 years old (mean age 6.7 years), participants’ height was measured to the nearest 0.1 cm using a Happened stadiometer, their weight measured to the nearest 0.1 kg using Tanita BC-558 Ironman Segmental Body Composition electronic scales and their SBP and DBP were measured twice with the child sitting and at rest with their arm supported, using a cuff size appropriate for their upper arm circumference using an Omron HEM 742 device (Omron Healthcare, Inc., Lake Forest, IL, USA).

In ALSPAC, birth weight was extracted from medical records, and birth length (crown-heel) was measured by ALSPAC staff who visited newborns soon after birth (median 1 day, range 1–14 days), using a Harpenden Neonatometer (Holtain Ltd, Crymych, Pembrokeshire, Wales, UK). For later outcomes, we analyse measures from research clinics held when participants were ∼7 (similar in age to the outcome measurements in the Pelotas 2004 cohort) and 18 years (oldest age of outcomes available, to see whether any associations persist into late adolescence). Mean ages at clinic attendence were 7.4 and 17.8 years. At each clinic, participants’ weight was measured to the nearest 0.1 kg using Tanita scales and height to the nearest 0.1 cm using a Harpenden stadiometer, both in light clothing and without shoes. SBP and DBP were measured twice with the participant sitting and at rest with their arm supported, using a cuff size appropriate for their upper arm circumference and an Omron IntelliSense M6 (Omron Healthcare, Kyoto, Japan). The mean of the two measures is used in our analyses.

### Other variables

Maternal and paternal educational levels were reported by the mother in antenatal or perinatal questionnaires/interviews in all cohorts. In ALSPAC, a questionnaire at 32 weeks gestation asked mothers to report their own and their partner’s educational attainment, which was categorised as below O-Level (Ordinary Level; exams taken in different subjects usually at age 15–16 at the completion of legally required school attendance, equivalent to today’s UK General Certificate of Secondary Education), O-Level only, A-Level (Advanced-Level; exams taken in different subjects usually at age 18) or university degree or above. Maternal schooling at the time of delivery for mothers of the Pelotas 2004 cohort was collected as a continuous variable and categorised according to the Brazilian Education System. The System is divided into three levels: fundamental (grades 1–8), intermediate^9, 10, 11^ and higher education (⩾12 years of formal education). Because of few numbers of women without any formal education (0 years) and with higher education, we opted to join these women with the nearest category available. In addition, we decided to split the 1–8 category because it is very common in the city for women to start the fundamental level and only complete 4 years. Finally, maternal education was categorised as 0–4, 5–8 and ⩾9 complete school years of formal education. Family income in Pelotas 2004 families in the month before delivery was expressed as minimum wage per month (standardized measure of income, one minimum wage was worth approximately US$ 80 in 2004). Parental occupation (reported by the mother in antenatal questionnaire) was used to derive household occupational social class in ALSPAC, with the highest of parental occupation used in all analyses.

### Statistical analysis

BMI was defined as weight in kilograms divided by height in metres squared. The association between birth order (first born compared with second born children (reference category)) and each outcome (birth weight, birth length, height, weight, BMI, SBP and DBP) was assessed using linear regression. Adjustment was initially made for gender and age at outcome assessment, and further models included control for confounders (maternal and paternal education in all cohorts, family income in Pelotas cohorts and household occupational social class in ALSPAC). Tests for gender interactions between the association between birth order and each outcome were conducted.

## Results

Of the 3669 children from the Pelotas 2004 cohort who were assessed at the 7-year follow-up, 932 were from families with two births and had complete data on all outcomes and confounders; 270 were first born (151 males) and 662 were second born (312 males). Of the 8290 participants attending the ALSPAC 7-year research clinic, 1385 were from families with two births and had data on all outcomes and confounders; 763 were first born children (372 males) and 622 were second born children (302 males). These participants form our eligible sample for analyses of outcomes from the 7-year clinic. Of the 5081 participants attending the ALSPAC 18-year research clinic, 1045 were from families with two births and had data on all outcomes and confounders; 586 were first born (268 males) and 459 were second born (199 males). These participants form our eligible sample for analyses of outcomes at 18 years.

Participants included in our analyses tended to be of higher socio-economic position compared with those excluded, but no large differences were seen in outcomes between included and excluded participants (Table 1; data shown only for participants included in analysis of 7-year outcomes, but findings for participants included in analysis of 18-year outcomes were similar; available from authors on request). No clear association between socio-economic position (as measured by maternal education) and family size was seen in ALSPAC, whereas in the Pelotas 2004 cohort one-child families were much more common in highly educated mothers, and larger (⩾3 children) families were much more common in mothers in the lowest education category (Supplementary Table 1).

In both cohorts, first born children were lighter and shorter at birth (Table 2). The association did not attenuate after adjustment for confounders. The associations of birth order with birth weight and length were slightly stronger in Pelotas compared with ALSPAC; for example, in the Pelotas 2004 cohort first born children had a birth weight that was on average 0.206 kg (95% CI: −0.283 to −0.129 kg) lighter than second born children after adjustment for gender and confounders, compared with ALSPAC where this difference was 0.142 kg (95% CI: −0.193 to −0.091 kg). However, confidence intervals for the estimated coefficients overlapped in the two cohorts for all outcomes.

Within the Pelotas 2004 cohort, there was some suggestion that first born children were shorter, lighter and with lower BMI, SBP and DBP at age 7 than second born children (Table 3). However, all coefficients were of small magnitude and confidence intervals were wide, for example, mean BMI difference comparing first to second born was −0.325 kg m^−2^ (95% CI: −0.725 to 0.074) after adjusting for confounders.

Within ALSPAC, there was no difference between first and second born children in height, weight or BMI at age 7 (Table 3). First born children tended to have slightly higher SBP and DBP, although coefficients were small and confidence intervals were wide. Associations remained similar with outcomes assessed at age 18 (Table 4).

In both cohorts, the conclusions for SBP and DBP remained similar when analyses were adjusted for height and BMI at the time of outcome assessment (Table 5).

For all analyses, there was no statistical evidence of gender interactions. The magnitude and direction of coefficients did seem to differ between males and females, but did not follow a consistent pattern, and these apparent differences most likely represent chance findings rather than any real differences. Gender-stratified results are presented in Supplementary Tables 2–5.

## Discussion

First born children tend to be of lower birth weight and shorter birth length than later born children. As low birth weight is associated with adverse cardiovascular risk in later life,^1, 2, 3^ it has been hypothesised that first born children may be at greater risk of adverse cardiovascular health than their later born siblings. In this study, despite first born children being shorter and smaller at birth, we did not observe any consistent associations between birth order and height, weight, BMI, SBP or DBP in a cohort born in 2004 in Pelotas, southern Brazil or in the ALSPAC cohort, born in 1991/1992 in Bristol, United Kingdom. Our analysis does not, therefore, support the hypothesis that first born children have greater weight, BMI and blood pressure relative to second born children.

Our findings are in contrast to some previous research. Analysis of the 1982 and 1993 birth cohorts from Pelotas (that is, the same city as the Pelotas 2004 cohort we analyse) has demonstrated that first born children are born smaller, but grow faster in infancy and are more adipose and with a more adverse cardiovascular profile in late adolescence/early adulthood.^4, 6^ In analysis of a subsample of the ALSPAC cohort, first born children were found to be over-represented in the group of infants experiencing ‘catch up growth’ in early infancy;^20^ our results suggest that any differences in postnatal growth do not result in longer term differences in body size or BP. While much of the research on birth order and adiposity and cardiovascular risk has been conducted in low- and middle-income countries,^4, 6, 8^ a small (*N*=85) study of children born in 2000–2007 in Auckland, New Zealand did find evidence that first born children had greater blood pressure and lower insulin sensitivity than later born children.^9^ The results of this study do not, however, have complete internal consistency; in addition to greater blood pressure and lower insulin sensitivity, first born children were thinner than later born children, which is contrary to the hypothesis and to other studies. Other, larger, studies in high-income settings have been less supportive of the hypothesis. One study involving over 1 million Swedish men found that first born children had slightly higher BMI but stronger muscle strength, and the authors observed no birth order differences in BP;^11^ other studies in Denmark,^12^ Japan^14^ and New Zealand^13^ have found no evidence that first born children are more likely to be overweight or have worse cardiovascular health. The studies in Denmark and Japan both found that only children and last-born children were at the greatest risk of obesity.^12, 14^

Two competing hypotheses for the putative association between birth order and cardiovascular health are that smaller birth size leads to adverse later health or that caring for, sharing with and competing against siblings generate differences in adiposity and cardiovascular health. If there was a single biological mechanism underlying the association between birth order and cardiovascular risk such that smaller birth size resulted in adverse changes to the cardiometabolic system, we might expect to see evidence of this association in diverse populations where the roles and responsibilities of first and second born children in households differ. However, if associations were explained by other mechanisms, for example, by first born children being expected to take care of their younger siblings and other household responsibilities, the association might potentially be more pronounced in cohorts from older generations compared with younger generations and in cohorts from lower- and middle-income settings compared with cohorts in higher income settings. This may be one reason underlying the differences between our findings and those from previous studies of low- and middle-income settings. However, one argument against the underlying mechanisms for the associations observed in previous studies being as a result of the expectations and pressure due to caring for younger siblings is that analysis of the 1993 Pelotas cohort identified differences in growth patterns comparing first and later born children that emerged in the first months of life, long before younger siblings had been born.^4^

Aspects of being an elder sibling (for example, caring responsibilities, rivalry, parenting differences) have been hypothesised to create birth order differences in personality, intelligence and other psychological traits.^15^ One quasi-experimental study using data from Norway showed that intelligence quotient (IQ) scores were highest in first born children, almost three units lower in second born children and another unit lower in third born children. However, second born children whose elder sibling died in infancy, and third born children whose two elder siblings both died in infancy, had IQ scores identical to first born children,^21^ suggesting that aspects of parenting rather than any intrauterine mechanism underlie the birth order differences in IQ. Other evidence, however, refutes the suggestion that birth order results in systematic differences in personality and other psychological traits, suggesting instead that parents respond to genetic variation in their children rather than generate differences through parenting.^22^

In contrast to previous studies, we restricted our analyses to families with two children in order to control for confounding by characteristics associated with family size. This distinction could underlie the difference in findings in this study compared with previous ones. Including higher birth order children in the analysis might increase the contrast in birth weight (and potentially other outcomes) between first and later born children, resulting in greater statistical power to detect the difference. It is also possible that studies including participants from larger families suffer from residual confounding by family size and characteristics of high birth order children. In our analyses, we found little evidence of social patterning in family size in ALSPAC, whereas in the Pelotas 2004 cohort larger family size was strongly associated with lower maternal education. Thus, it is possible that the differences between first and later born children observed in previous analyses of older cohorts from Pelotas arise mainly within larger, poorer families. Alternatively, birth order may operate differently depending on family size. This is plausible if the mechanism is primarily due to the pressures associated with being a first born child, as these pressures may be greater if there are a larger number of younger siblings with whose care the first born is expected to be involved.

A key strength of our analysis is the comparison between two cohorts from very different settings, which enables greater strength of inference than is possible from a single cohort. In both cohorts, we had a sufficient sample size to be able to restrict our analyses to families with two children in order to control for family size, and outcomes were measured both at birth and in mid-childhood. An important limitation of the analyses using the Pelotas 2004 cohort is that we only have data on siblings born up to when study participants were of age 4 years. Some families would have gone on to have additional siblings, meaning that these families would have been excluded from our analyses due to having more than two children.

In conclusion, our findings do not support an association between birth order and cardiovascular risk when comparing first and second born offspring. No systematic difference between first born and second born children was observed for height, weight, BMI, SBP or DBP in either the Pelotas 2004 cohort or the ALSPAC. Reasons for the differences between our findings and those from previous studies could include differences in populations resulting in differences in the mechanisms that could be hypothesised to underlie associations and residual confounding by characteristics associated with family size in previous studies.

## Figures and Tables

**Table 1:** Number (%) or mean (s.d.) of participant characteristics for those included and excluded from our analyses at 7-year follow-up

*Pelotas 2004 cohort*	*Included participants*	*Excluded participants*
	N*=932*	N *depends on variable*
Male offspring	463 (49.7%)	1759 (52.5%)
		
*Maternal education*		
0–4 years	107 (11.5%)	558 (16.9%)
5–8 years	398 (42.7%)	1360 (41.1%)
9 or more years	427 (45.8%)	1390 (42.0%)
		
*Paternal education*		
0–4 years	134 (14.4%)	451 (18.8%)
5–8 years	349 (37.5%)	831 (34.7%)
9 or more years	449 (48.2%)	1115 (46.5%)
Birth weight (kg)	3.21 (0.55)	3.15 (0.53) *N*=3255
Birth length (cm)	48.29 (2.56)	48.15 (2.61) *N*=3236
Height at 7-year clinic (cm)	121.26 (5.67)	120.78 (5.69) *N*=2720
Weight at 7-year clinic (kg)	25.17 (5.96)	24.87 (6.00) *N*=2763
BMI at 7-year clinic (kg m^−2^)	16.97 (2.94)	16.90 (2.92) *N*=2718
SBP at 7-year clinic (mmHg)	99.10 (9.31)	99.17 (9.81) *N*=2641
DBP at 7-year clinic (mmHg)	60.46 (8.58)	60.51 (8.84) *N*=2640
*ALSPAC—participants included in analysis of 7-year outcomes*	N*=1385*	N *depends on variable*
Male offspring	674 (48.7%)	9403 (52.0%)
		
*Maternal education*		
<O-Level	168 (12.1%)	3585 (32.3%)
O-Level	485 (35.0%)	3845 (34.6%)
A-Level	422 (30.5%)	2381 (21.4%)
Degree or above	310 (22.4%)	1297 (11.7%)
		
*Paternal education*		
<O-Level	274 (19.8%)	3884 (36.6%)
O-Level	302 (21.8%)	2250 (21.2%)
A-Level	414 (29.9%)	2707 (25.5%)
Degree or above	395 (28.5%)	1786 (16.8%)
		
*Household social class*		
I (highest)	282 (20.4%)	1257 (12.3%)
II	670 (48.4%)	4167 (40.9%)
III non-manual	317 (22.9%)	2630 (25.8%)
III manual	99 (7.2%)	1470 (14.4%)
IV or V (lowest)	17 (1.2%)	668 (6.6%)
Birth weight (kg)	3.44 (0.49)	3.38 (0.59) *N*=12 516
Birth length (cm)	50.77 (2.34)	50.54 (2.58) *N*=9912
Height at 7-year clinic (cm)	125.78 (5.26)	125.94 (5.73) *N*=6839
Weight at 7-year clinic (kg)	25.48 (4.13)	26.01 (4.86) *N*=6826
BMI at 7-year clinic (kg m^−2^)	16.04 (1.89)	16.31 (2.14) *N*=6825
SBP at 7-year clinic (mmHg)	98.40 (9.06)	99.18 (9.32) *N*=6782
DBP at 7-year clinic (mmHg)	56.14 (6.41)	56.63 (6.75) *N*=6782

Abbreviations: BMI, body mass index; DBP, diastolic blood pressure; SBP, systolic blood pressure.

**Table 2:** Association between birth order and birth size in each cohort

	*Birth weight (kg)*	*Birth length (cm)*
*Pelotas 2004 cohort,* N*=932*		
Gender adjusted	−0.221 (−0.297 to −0.145) *P*<0.001	−0.992 (−1.347 to −0.637) *P*<0.001
Gender and confounder adjusted	−0.206 (−0.283 to −0.129) *P*<0.001	−0.931 (−1.289 to −0.574) *P*<0.001
		
*ALSPAC,* N*=1385*		
Gender adjusted	−0.140 (−0.191 to −0.089) *P*<0.001	−0.406 (−0.649 to −0.164) *P*=0.001
Gender and confounder adjusted	−0.142 (−0.193 to −0.091) *P*<0.001	−0.421 (−0.664 to −0.179) *P*<0.001

Abbreviations: BMI, body mass index; DBP, diastolic blood pressure; SBP, systolic blood pressure.

Second born is the reference category; coefficients are mean difference comparing first with second born from linear regressions. Confounders are maternal and paternal education and family income for Pelotas 2004, maternal and paternal education and highest occupational social class in the household for ALSPAC. In all, 270 Pelotas participants were first born and 662 were second born. In all, 763 ALSPAC participants were first born children and 622 were second born.

**Table 3:** Association between birth order and later height, weight, BMI, SBP and DBP in each cohort

	*Height (cm)*	*Weight (kg)*	*BMI (kg m^−2^)*	*SBP (mmHg)*	*DBP (mmHg)*
*Pelotas 2004 cohort,* N*=932*					
Gender and age adjusted	−0.160 (−0.910 to 0.590) *P*=0.68	−0.476 (−1.278 to 0.326) *P*=0.25	−0.304 (−0.702 to 0.095) *P*=0.14	−0.121 (−1.410 to 1.167) *P*=0.85	−0.408 (−1.594 to 0.777) *P*=0.50
Gender, age and confounder adjusted	−0.202 (−0.941 to 0.538) *P*=0.59	−0.530 (−1.328 to 0.267) *P*=0.19	−0.325 (−0.725 to 0.074) *P*=0.11	−0.143 (−1.443 to 1.157) *P*=0.83	−0.401 (−1.597 to 0.795) *P*=0.51
					
*ALSPAC,* N*=1385*					
Gender and age adjusted	0.003 (−0.546 to 0.552) *P*=0.99	−0.065 (−0.501 to 0.371) *P*=0.77	−0.043 (−0.243 to 0.158) *P*=0.68	0.074 (−0.887 to 1.034) *P*=0.88	0.534 (−0.144 to 1.211) *P*=0.12
Gender, age and confounder adjusted	−0.035 (−0.584 to 0.515) *P*=0.90	−0.080 (−0.516 to 0.356) *P*=0.72	−0.043 (−0.243 to 0.158) *P*=0.68	0.104 (−0.857 to 1.066) *P*=0.83	0.513 (−0.164 to 1.191) *P*=0.14

Abbreviations: BMI, body mass index; DBP, diastolic blood pressure; SBP, systolic blood pressure.

Second born is the reference category; coefficients are mean difference comparing first with second born from linear regressions. Mean age at follow-up is 6.7 years in Pelotas 2004, 7.4 years in ALSPAC. Confounders are maternal and paternal education and family income for Pelotas 2004, maternal and paternal education and highest occupational social class in the household for ALSPAC. In all, 270 Pelotas participants were first born and 662 were second born. In all, 763 ALSPAC participants were first born children and 622 were second born.

**Table 4:** Association between birth order and later height, weight, BMI, SBP and DBP at 18 years in ALSPAC

	*Height (cm)*	*Weight (kg)*	*BMI (kg m*^−2^)	*SBP (mmHg)*	*DBP (mmHg)*
*ALSPAC,* N*=1045*					
Gender and age adjusted	−0.486 (−1.249 to 0.277) *P*=0.21	−0.106 (−1.538 to 1.327) *P*=0.89	0.105 (−0.339 to 0.549) *P*=0.64	0.646 (−0.464 to 1.756) *P*=0.25	0.329 (−0.451 to 1.109) *P*=0.41
Gender, age and confounder adjusted	−0.500 (−1.261 to 0.262) *P*=0.20	−0.134 (−1.564 to 1.295) *P*=0.85	0.099 (−0.345 to 0.542) *P*=0.66	0.659 (−0.451 to 1.769) *P*=0.24	0.316 (−0.463 to 1.094) *P*=0.43

Abbreviations: BMI, body mass index; DBP, diastolic blood pressure; SBP, systolic blood pressure.

Second born is the reference category; coefficients are mean difference comparing first with second born from linear regressions. Mean age at follow-up is 17.7 years. Confounders are maternal and paternal education and highest occupational social class in the household. In all, 586 participants were first born children and 459 were second born.

**Table 5:** Association between birth order and SBP and DBP at age 7 in each cohort, with and without adjustment for height and BMI at the time of BP assessment

	*SBP (mmHg)*	*DBP (mmHg)*
*Pelotas 2004 cohort,* N*=932*		
Gender, age and confounder adjusted	−0.143 (−1.443 to 1.157) *P*=0.83	−0.401 (−1.597 to 0.795) *P*=0.51
Adjusted for gender, age, confounders, height and BMI at age 7	0.178 (−1.070 to 1.426) *P*=0.78	−0.205 (−1.399 to 0.990) *P*=0.74
		
*ALSPAC,* N*=1385*		
Gender, age and confounder adjusted	0.104 (−0.857 to 1.066) *P*=0.83	0.513 (−0.164 to 1.191) *P*=0.14
Adjusted for gender, age, confounders, height and BMI at age 7	0.168 (−0.732 to 1.068) *P*=0.71	0.535 (−0.133 to 1.203) *P*=0.12

Abbreviations: BMI, body mass index; DBP, diastolic blood pressure; SBP, systolic blood pressure.

Second born is the reference category; coefficients are mean difference comparing first with second born from linear regressions. Mean age at follow-up is 6.7 years in Pelotas 2004, 7.4 years in ALSPAC. Confounders are maternal and paternal education and family income for Pelotas 2004, maternal and paternal education and highest occupational social class in the household for ALSPAC. In all, 270 Pelotas participants were first born and 662 were second born. In all, 763 ALSPAC participants were first born children and 622 were second born.

## References

[bib1] Barker DJ, Gluckman PD, Godfrey KM, Harding JE, Owens JA, Robinson JS. Fetal nutrition and cardiovascular disease in adult life. Lancet 1993; 341: 938–941. 8096277 10.1016/0140-6736(93)91224-a

[bib2] Barker DJP, Osmond C, Forsen TJ, Kajantie E, Eriksson JG. Trajectories of growth among children who have coronary events as adults. N Engl J Med 2005; 353: 1802–1809. 16251536 10.1056/NEJMoa044160

[bib3] Eriksson JG, Forsen T, Tuomilehto J, Osmond C, Barker DJP. Early growth and coronary heart disease in later life: longitudinal study. Br Med J 2001; 322: 949–953. 11312225 10.1136/bmj.322.7292.949 PMC31033

[bib4] Wells JC, Hallal PC, Reichert FF, Dumith SC, Menezes AM, Victora CG. Associations of birth order with early growth and adolescent height, body composition, and blood pressure: prospective birth cohort from Brazil. Am J Epidemiol 2011; 174: 1028–1035. 21940799 10.1093/aje/kwr232 PMC3658103

[bib5] Rosenberg M. Birth weights in three Norwegian cities, 1860–1984. Secular trends and influencing factors. Ann Hum Biol 1988; 15: 275–288. 3044272 10.1080/03014468800009751

[bib6] Siervo M, Horta BL, Stephan BC, Victora CG, Wells JC. First-borns carry a higher metabolic risk in early adulthood: evidence from a prospective cohort study. PLoS One 2010; 5: e13907. 21085691 10.1371/journal.pone.0013907 PMC2976719

[bib7] Ghosh JR, Bandyopadhyay AR. Income, birth order, siblings, and anthropometry. Hum Biol 2006; 78: 733–741. 17564251 10.1353/hub.2007.0012

[bib8] Dahly DL, Adair LS. Does lower birth order amplify the association between high socioeconomic status and central adiposity in young adult Filipino males? Int J Obes 2010; 34: 751–759. 10.1038/ijo.2009.275 PMC2908417 20065964

[bib9] Ayyavoo A, Savage T, Derraik JG, Hofman PL, Cutfield WS. First-born children have reduced insulin sensitivity and higher daytime blood pressure compared to later-born children. J Clin Endocrinol Metab 2013; 98: 1248–1253. 23365122 10.1210/jc.2012-3531

[bib10] Siervo M, Stephan BC, Colantuoni A, Wells JC. First-borns have a higher metabolic rate and carry a higher metabolic risk in young women attending a weight loss clinic. Eat Weight Disord 2011; 16: e171–e176. 22290032 10.1007/BF03325128

[bib11] Jelenkovic A, Silventoinen K, Tynelius P, Myrskyla M, Rasmussen F. Association of birth order with cardiovascular disease risk factors in young adulthood: a study of one million Swedish men. PLoS One 2013; 8: e63361. 23696817 10.1371/journal.pone.0063361 PMC3656047

[bib12] Haugaard LK, Ajslev TA, Zimmermann E, Angquist L, Sorensen TI. Being an only or last-born child increases later risk of obesity. PLoS One 2013; 8: e56357. 23437116 10.1371/journal.pone.0056357 PMC3577826

[bib13] Savage T, Derraik JG, Miles HL, Mouat F, Cutfield WS, Hofman PL. Birth order progressively affects childhood height. Clin Endocrinol 2013; 79: 379–385. 10.1111/cen.12156 23347499

[bib14] Ochiai H, Shirasawa T, Ohtsu T, Nishimura R, Morimoto A, Obuchi R et al. Number of siblings, birth order, and childhood overweight: a population-based cross-sectional study in Japan. BMC Public Health 2012; 12: 766. 22966779 10.1186/1471-2458-12-766 PMC3509397

[bib15] Sulloway FJ. Born to rebel. Birth order, family dynamics and creative lives. Abacus: London, UK, 1996.

[bib16] Santos IS, Barros AJ, Matijasevich A, Domingues MR, Barros FC, Victora CG. Cohort profile: the 2004 Pelotas (Brazil) birth cohort study. Int J Epidemiol 2011; 40: 1461–1468. 20702597 10.1093/ije/dyq130 PMC3235016

[bib17] Barros AJ, da Silva dS I, Victora CG, Albernaz EP, Domingues MR, Timm IK et al. The 2004 Pelotas birth cohort: methods and description. Rev Saude Publica 2006; 40: 402–413. 16810363 10.1590/s0034-89102006000300007

[bib18] Boyd A, Golding J, Macleod J, Lawlor DA, Fraser A, Henderson J et al. Cohort Profile: The ‘Children of the 90s’-the index offspring of the Avon Longitudinal Study of Parents and Children. Int J Epidemiol 2012; 42: 111–127. 22507743 10.1093/ije/dys064 PMC3600618

[bib19] Fraser A, Macdonald-Wallis C, Tilling K, Boyd A, Golding J, Davey SG et al. Cohort Profile: The Avon Longitudinal Study of Parents and Children: ALSPAC mothers cohort. Int J Epidemiol 2012; 42: 97–110. 22507742 10.1093/ije/dys066 PMC3600619

[bib20] Ong KK, Ahmed ML, Emmett PM, Preece MA, Dunger DB. Association between postnatal catch-up growth and obesity in childhood: prospective cohort study. Br Med J 2000; 320: 967–971. 10753147 10.1136/bmj.320.7240.967 PMC27335

[bib21] Kristensen P, Bjerkedal T. Explaining the relation between birth order and intelligence. Science 2007; 316: 1717. 17588924 10.1126/science.1141493

[bib22] Harris JR. No two alike: human nature and human individuality. WW Norton Company: New York, NY, USA, 2006.

