# Supplementary material for: The association of birth order with later body mass index and blood pressure: a comparison between prospective cohort studies from the United Kingdom and Brazil
Source: Int J Obes (Lond). 2013 Oct 29;38(7):973–9. doi: 10.1038/ijo.2013.189 (PMC4024316; doi:10.1038/ijo.2013.189)
Supplement: Supplementary Tables [file ijo2013189x1.doc]

**Online-only supplementary material for:**

**The association of birth order with later body mass index and blood pressure: a comparison between prospective cohort studies from the UK and Brazil**

Laura D Howe*, Pedro C Hallal, Alicia Matijasevich, Jonathan C Wells, Iná S. Santos, Aluísio J. D. Barros, Debbie A Lawlor, Cesar G Victora, George Davey Smith

**Affiliations:** MRC Integrative Epidemiology Unit at the University of Bristol, Bristol, UK (Laura D Howe, Debbie A Lawlor, George Davey Smith); School of Social and Community Medicine, University of Bristol, Bristol, UK (Laura D Howe, Debbie A Lawlor, George Davey Smith); Postgraduate Programme in Epidemiology, Federal University of Pelotas, Pelotas, Brazil (Pedro C Hallal, Alicia Matijasevich, Iná S. Santos, Aluísio J. D. Barros, Cesar G Victora); Institute of Child Health, University College London, London, UK (Jonathan C Wells)

*Address for correspondence: Laura D Howe, MRC Integrative Epidemiology Unit at the University of Bristol, School of Social and Community Medicine, Oakfield House, Oakfield Grove, University of Bristol, Bristol BS8 2BN, UK. Email: Laura.Howe@bristol.ac.uk. Telephone: +44 117 3310134

**Supplementary Table 1: The association between family size and maternal education**

|  | **Family size (number of children)** | | |  |
| --- | --- | --- | --- | --- |
|  | **1 child** | **2 children** | **3 or more children** | P value from  chi-2 test |
| ***Pelotas 2004 cohort, N=3,762*** |  |  |  |  |
| Maternal education |  |  |  |  |
| *0-4 years* | 100 (17.5%) | 160 (28.1%) | 310 (54.4%) |  |
| *5-8 years* | 486 (31.0%) | 580 (37.0%) | 500 (31.9%) |  |
| *9 or more years* | 812 (49.9%) | 591 (36.4%) | 223 (13.7%) | <0.001 |
|  |  |  |  |  |
| ***ALSPAC, N=3,760*** |  |  |  |  |
| Maternal education |  |  |  |  |
| *< O-Level* | 77 (13.1%) | 293 (49.8%) | 219 (37.2%) |  |
| *O-Level* | 156 (12.1%) | 708 (55.1%) | 422 (32.8%) |  |
| *A-Level* | 159 (14.5%) | 601 (54.8%) | 337 (30.7%) |  |
| *Degree or above* | 93 (11.8%) | 439 (55.7%) | 256 (32.5%) | 0.08 |

In Pelotas, family size is calculated based on information on the number of siblings reported in the parent-completed questionnaire when the index child was aged 4 years. We include all participants with non-missing data on maternal education and number of siblings.

In ALSPAC, family size is calculated based on information on the number and outcome of pregnancies reported in questionnaires at enrollment into the study, when the index child was aged 7 years, and when the index child was aged 18 years. We include all participants with data from these three questionnaires and non-missing data on maternal education.

Supplementary Table 2: Association between birth order and birth size in each cohort; stratified by gender

|  | **Birth weight (kg)** | **Birth length (cm)** |
| --- | --- | --- |
| ***Pelotas 2004 cohort*** |  |  |
| Males: unadjusted | -0.274 (-0.381 to -0.167)  P<0.001 | -1.169 (-1.662 to -0.677)  P<0.001 |
| Males: confounder adjusted | -0.265 (-0.372 to -0.158)  P<0.001 | -1.139 (-1.632 to -0.646)  P<0.001 |
| Females: unadjusted | -0.161 (-0.270 to -0.051)  P=0.004 | -0.789 (-1.302 to -0.277)  P=0.003 |
| Females: confounder adjusted | -0.137 (-0.248 to -0.026)  P=0.02 | -0.674 (-1.196 to -0.152)  P=0.01 |
|  |  |  |
| ***ALSPAC*** |  |  |
| Males: unadjusted | -0.164 (-0.242 to -0.085) p<0.001 | -0.463 (-0.819 to -0.107) p=0.01 |
| Males: confounder adjusted | -0.163 (-0.241 to -0.086) p<0.001 | -0.479 (-0.834 to -0.124) p=0.01 |
| Females: unadjusted | -0.117 (-0.184 to -0.051) p=<0.001 | -0.353 (-0.684 to -0.022) p=0.04 |
| Females: confounder adjusted | -0.114 (-0.181 to -0.047) p<0.001 | -0.354 (-0.686 to -0.021) p=0.04 |

Second born is the reference category; coefficients are mean difference comparing first with second born from linear regressions. Confounders are maternal and paternal education and family income for Pelotas 2004, maternal and paternal education and highest occupational social class in the household for ALSPAC. 270 Pelotas participants were first born and 662 were second born. 763 ALSPAC participants were first born children and 622 were second born.

BMI: body mass index. cm: centimeters. DBP: diastolic blood pressure. kg: kilograms. mmHG: millimetres of mercury. SBP: systolic blood pressure.

**Table 3: Association between birth order and later height, weight, BMI, SBP and DBP in each cohort; stratified by gender**

|  | **Height (cm)** | **Weight (kg)** | **BMI (kg/m^2^)** | **SBP (mmHg)** | **DBP (mmHg)** |
| --- | --- | --- | --- | --- | --- |
| ***Pelotas 2004 cohort*** |  |  |  |  |  |
| Males: age- adjusted | -0.011 (-1.022 to 1.001) p=0.98 | -0.048 (-1.047 to 0.951) p=0.93 | -0.058 (-0.548 to 0.433) p=0.818 | 0.706 (-1.057 to 2.469) p=0.43 | 0.150 (-1.486 to 1.785) p=0.86 |
| Males: confounder adjusted | -0.191 (-1.174 to 0.793) p=0.70 | -0.174 (-1.160 to 0.812) p=0.729 | -0.091 (-0.581 to 0.399) p=0.72 | 0.642 (-1.127 to 2.412) p=0.48 | 0.123 (-1.520 to 1.766) p=0.883 |
| Females: age- adjusted | -0.331 (-1.450 to 0.787) p=0.56 | -0.972 (-2.248 to 0.304) p=0.14 | -0.590 (-1.230 to 0.050) p=0.071 | -1.083 (-2.971 to 0.805) p=0.26 | -1.061 (-2.787 to 0.664) p=0.23 |
| Females: confounder adjusted | -0.137 (-1.255 to 0.981) p=0.81 | -0.886 (-2.167 to 0.395) p=0.18 | -0.580 (-1.225 to 0.065) p=0.08 | -0.979 (-2.901 to 0.943) p=0.32 | -1.033 (-2.791 to 0.726) p=0.25 |
|  |  |  |  |  |  |
| ***ALSPAC*** |  |  |  |  |  |
| Males: age- adjusted | 0.511 (-0.283 to 1.305) p=0.21 | 0.058 (-0.587 to 0.703) p=0.86 | -0.277 (-1.665 to 1.111) p=0.70 | -0.277 (-1.665 to 1.111) p=0.70 | 0.877 (-0.094 to 1.847) p=0.08 |
| Males: confounder adjusted | 0.449 (-0.345 to 1.244) p=0.27 | 0.032 (-0.615 to 0.679) p=0.92 | -0.259 (-1.651 to 1.133) p=0.72 | -0.259 (-1.651 to 1.133) p=0.72 | 0.839 (-0.133 to 1.811) p=0.09 |
| Females: age- adjusted | -0.439 (-1.199 to 0.322) p=0.26 | -0.148 (-0.739 to 0.444) p=0.63 | 0.433 (-0.902 to 1.768) p=0.53 | 0.433 (-0.902 to 1.768) p=0.53 | 0.219 (-0.732 to 1.169) p=0.65 |
| Females: confounder adjusted | -0.444 (-1.205 to 0.318) p=0.25 | -0.144 (-0.736 to 0.448) p=0.63 | 0.491 (-0.847 to 1.829) p=0.47 | 0.491 (-0.847 to 1.829) p=0.47 | 0.213 (-0.739 to 1.165) p=0.66 |
|  |  |  |  |  |  |

Second born is the reference category; coefficients are mean difference comparing first with second born from linear regressions. Mean age at follow up is 6.7 years in Pelotas 2004, 7.4 years in ALSPAC. Confounders are maternal and paternal education and family income for Pelotas 2004, maternal and paternal education and highest occupational social class in the household for ALSPAC. 270 Pelotas participants were first born and 662 were second born. 763 ALSPAC participants were first born children and 622 were second born.

BMI: body mass index. cm: centimeters. DBP: diastolic blood pressure. kg: kilograms. mmHG: millimetres of mercury. SBP: systolic blood pressure.

**Supplementary** Table 4: Association between birth order and later height, weight, BMI, SBP and DBP at 18 years in ALSPAC; stratified by gender

|  | **Height (cm)** | **Weight (kg)** | **BMI (kg/m^2^)** | **SBP (mmHg)** | **DBP (mmHg)** |
| --- | --- | --- | --- | --- | --- |
| ***ALSPAC*** |  |  |  |  |  |
| Males: age- adjusted | -0.542 (-1.303 to 0.219) p=0.16 | -0.069 (-1.498 to 1.361) p=0.93 | 0.134 (-0.307 to 0.575) p=0.55 | 0.703 (-0.404 to 1.809) p=0.21 | 0.379 (-0.397 to 1.155) p=0.34 |
| Males: confounder adjusted | 0.201 (-1.003 to 1.405) p=0.74 | -0.351 (-2.816 to 2.115) p=0.78 | -0.157 (-0.862 to 0.547) p=0.66 | -0.177 (-1.927 to 1.572) p=0.84 | -0.206 (-1.387 to 0.974) p=0.73 |
| Females: age- adjusted | 0.105 (-1.100 to 1.309) p=0.87 | -0.292 (-2.774 to 2.189) p=0.82 | -0.115 (-0.822 to 0.591) p=0.75 | -0.199 (-1.949 to 1.551) p=0.82 | -0.167 (-1.353 to 1.019) p=0.78 |
| Females: confounder adjusted | -1.060 (-2.032 to -0.088) p=0.03 | 0.038 (-1.615 to 1.692) p=0.96 | 0.304 (-0.262 to 0.869) p=0.29 | 1.327 (-0.095 to 2.748) p=0.07 | 0.732 (-0.305 to 1.769) p=0.17 |
|  | -1.088 (-2.063 to -0.114) p=0.03 | 0.117 (-1.531 to 1.765) p=0.89 | 0.341 (-0.220 to 0.902) p=0.23 | 1.374 (-0.042 to 2.790) p=0.06 | 0.776 (-0.253 to 1.805) p=0.14 |

Second born is the reference category; coefficients are mean difference comparing first with second born from linear regressions. Mean age at follow up is 17.7 years. Confounders are maternal and paternal education and highest occupational social class in the household. 586 participants were first born children and 459 were second born.

BMI: body mass index. cm: centimeters. DBP: diastolic blood pressure. kg: kilograms. mmHG: millimetres of mercury. SBP: systolic blood pressure.

**Suppementary Table 5: Association between birth order and SBP and DBP at age 7 in each cohort, with and without adjustment for height and BMI at the time of BP assessment; stratified by gender**

|  | **SBP (mmHg)** | **DBP (mmHg)** |
| --- | --- | --- |
| ***Pelotas 2004 cohort*** |  |  |
| Males: confounder adjusted | 0.642 (-1.127 to 2.412) p=0.48 | 0.123 (-1.520 to 1.766) p=0.883 |
| Males: Adjusted for confounders, height and BMI at age 7 | 1.258 (-0.451 to 2.967)  P=0.15 | 0.574 (-1.060 to 2.208)  P=0.49 |
| Females: confounder adjusted | -0.979 (-2.901 to 0.943) p=0.32 | -1.033 (-2.791 to 0.726) p=0.25 |
| Females: Adjusted for confounders, height and BMI at age 7 | -1.065 (-2.903 to 0.774)  P=0.26 | -1.095 (-2.859 to 0.668)  P=0.22 |
|  |  |  |
| ***ALSPAC*** |  |  |
| Males: confounder adjusted | -0.259 (-1.651 to 1.133) p=0.72 | 0.839 (-0.133 to 1.811) p=0.09 |
| Males: Adjusted for confounders, height and BMI at age 7 | -0.262 (-1.570 to 1.046)  P=0.69 | 0.812 (-0.133 to 1.203)  P=0.10 |
| Females: confounder adjusted | 0.491 (-0.847 to 1.829) p=0.47 | 0.213 (-0.739 to 1.165) p=0.66 |
| Females: Adjusted for confounders, height and BMI at age 7 | 0.622 (-0.631 to 1.875)  P=0.33 | 0.235 (-0.705 to 1.176)  P=0.62 |

Second born is the reference category; coefficients are mean difference comparing first with second born from linear regressions. Mean age at follow up is 6.7 years in Pelotas 2004, 7.4 years in ALSPAC. Confounders are maternal and paternal education and family income for Pelotas 2004, maternal and paternal education and highest occupational social class in the household for ALSPAC. 270 Pelotas participants were first born and 662 were second born. 763 ALSPAC participants were first born children and 622 were second born.
